# Ferruginated Pill of Mercury

**Published:** 1843-08

**Authors:** G. F. Collier


*Ferruginated Pill of Mercury.* By G. F. Collier, M.D.—The varying and unsatisfactory quality of our "blue-pill" has for many years attracted attention. In my second edition of the "London Pharmacopœia" I gave the outline of a formula for preparing it with sesquioxide of iron; the further experience of years enables me to offer this preparation to the profession as a boon to them and to the public, for it may be proved that the iron enters along with the mercury into the blood, and saves the wear and tear of the human body under its use. I will not, at this time canvass, because I doubt, the probability of the sesquioxide (in double equivalents) yielding up one atom of its oxygen to the mercury, as is the opinion of several chemists to whom I have submitted this compound. I am aware that other peroxides, when moist, will similarly combine and divide mercurial globules; and I will not now enter upon the series of incomplete experiments instituted by myself to prove that mercury will amalgamate with other metallic oxides, and in this state form double or triple salts with acids. I shall now keep only to the practical utility of my compound, and to its importance as a great remedial improvement.

In the spirit of a pharmaceutical chemist I should either exclusively manufacture and sell it over my own counter, or I should take out a patent for it; in that of a manufacturing chemist I should do the like, or keep it a secret and save a fortune by it; were I of the new school of "summi auctores" I should throw the mere shadow of it, chemical, mesmerical, magnetical, and homœopathical, into a modest pamphlet, proving that the era of medicine truly commenced in 1843, and that up to that time all were shadows, clouds, and darkness. I shall content myself with publishing the recipe, and recording a brief statement of my experience.

℞.—Ferri sesquioxydi, ʒi;

Hydrargyri, ʒij;

Confect. rosæ Gallicæ, ʒiij. Contere donec globuli non amplius conspiciantur.

It is made in five minutes; common blue-pill demands a week. The globules are not visible, even by the microscope. It is uniform in its appearance and effects. It makes a smoother pill, retaining its form more permanently. It salivates in a few days in the usual doses. The presence of the iron prevents the wear and tear of the human body under the effects of the mercury. It is particularly eligible for the strumous, the irritable, and for reduced anemial constitutions requiring mercury. The powers of life are not so much (scarcely at all) prostrated under its use. Its resolvent power is greater than that of mercury alone, especially with respect to buboes. Practitioners will at all events know what they are using; at present they have for blue-pill all manner of alloys and sulphurets,—murcurial zinc pill, mercurial-sulphur pill, &c., &c., &c.

Five grains of sesquioxide will suffice to amalgamate and divide a large quantity of mercury, but I propose the larger proportion as a remedy.—*Lancet*, March 11, 1843.

